# Effectiveness of EMDR in patients with substance use disorder and comorbid PTSD: study protocol for a randomized controlled trial

**DOI:** 10.1186/s12888-017-1255-9

**Published:** 2017-03-16

**Authors:** Ingo Schäfer, Laycen Chuey-Ferrer, Arne Hofmann, Peter Lieberman, Günter Mainusch, Annett Lotzin

**Affiliations:** 1 Department of Psychiatry and Psychotherapy, University Medical Center Hamburg-Eppendorf, Hamburg, Germany; 2 Center for Interdisciplinary Addiction Research, Department of Psychiatry and Psychotherapy, University Medical Center Hamburg-Eppendorf, University of Hamburg, Martinistr. 52, Hamburg, D-20246 Germany; 3 AHG Clinic Dormagen, Dormagen, Germany; 4 EMDR-Institute Germany, Bergisch Gladbach, Germany

**Keywords:** Posttraumatic stress disorder, Alcohol, Substance abuse, Addiction, Comorbidity, Eye movement desensitization and reprocessing, Trauma-focused treatment, Exposure-based treatment, Psychotherapy outcome research

## Abstract

**Background:**

Eye Movement Desensitization and Reprocessing (EMDR) is an evidence-based treatment for PTSD. However, it is unclear whether EMDR shows the same effectiveness in patients with substance use disorders (SUD) and comorbid PTSD. In this trial, we examine the effectiveness of EMDR in reducing PTSD symptoms in patients with SUD and PTSD.

**Methods/Design:**

We conduct a single-blinded RCT among 158 patients with SUD and comorbid PTSD admitted to a German addiction rehabilitation center specialized for the treatment of patients with SUD and comorbid PTSD. Patients are randomized to receive either EMDR, added to SUD rehabilitation and non-trauma-focused PTSD treatment (TAU), or TAU alone. The primary outcome is change from baseline in PTSD symptom severity as measured by the Clinician-Administered PTSD Scale at 6-month follow-up. Secondary outcomes are change from baseline in substance use, addiction-related problems, depressive symptoms, dissociative symptoms, emotion dysregulation and quality of life. Assessments are carried out by blinded raters at admission, at end of treatment, and at 3- and 6-month follow-up. We expect that EMDR plus TAU will be more effective in reducing PTSD symptoms than TAU alone. Mixed models will be conducted using an intention-to-treat and per-protocol approach.

**Discussion:**

This study aims to expand the knowledge about the effectiveness of EMDR in patients with SUD and comorbid PTSD. The expected finding of the superiority of EMDR in reducing PTSD symptoms compared to non-trauma-focused PTSD treatment may enhance the use of trauma-focused treatment approaches for patients with SUD and co-morbid PTSD.

**Trial registration:**

German Clinical Trials Register: DRKS00009007; U1111-1172-9213. Retrospectively registered 01 Juni 2016.

## Background

Up to 45% of the patients with SUD experience comorbid PTSD [1–3], indicating a clear need for PTSD treatment in this population. Although the importance of comorbid PTSD in the treatment of patients with SUD has been recognized [4], most SUD inpatient rehabilitation centers in Germany do not offer integrated treatment for SUD and PTSD. When an integrated treatment is offered, non-trauma-focused interventions are predominantly used to address PTSD [5]. This current clinical practice in routine SUD healthcare is in contrast to the national and international guidelines [6–8] that recommend trauma-focused treatment for PTSD, which clearly yields higher effect sizes compared with non-trauma-focused approaches. However, the evidence base of these guidelines is built on trials in which patients with PTSD and comorbid SUD have been almost continuously excluded [9, 10].

First evidence exists that trauma-focused treatments, such as prolonged exposure [11], trauma-focused imaginal exposure [12] and structured writing therapy [13], may also be effective in patients with PTSD and comorbid SUD in reducing PTSD symptoms, although the evidence is not consistent [13–15]. This trials also found that trauma-focused interventions for patients with SUD and comorbid PTSD could be applied securely without compromising substance use outcomes [14, 15]. This is an important result, because clinicians may hesitate to use trauma-focused therapy for patients with SUD and comorbid PTSD, because they might believe that eliciting intense emotions related to the traumatic event during trauma-focused treatment may increase the risk for relapse [16, 17]. EMDR is another trauma-focused treatment that has been shown to be effective in patients with PTSD alone [6, 18]. So far, no RCT has been conducted that examined whether EMDR is effective in reducing PTSD symptoms in patients with SUD and comorbid PTSD.

Trauma-focused PTSD treatment among patients with SUD may also effectively reduce SUD symptoms [19, 20]. Patients with PTSD and SUD report higher levels of craving in response to trauma-related cues [21] than patients with PTSD. Therefore, these patients might be more likely to use substances to regulate negative affective states associated with PTSD [20, 22, 23]. If negative affective states related to PTSD could be effectively reduced by trauma-focused treatment, SUD symptoms might also be reduced.

### Research aims and hypothesis

The primary aim of this study is to investigate the effectiveness of EMDR, added to SUD rehabilitation and non-trauma-focused PTSD treatment (TAU), in reducing PTSD symptom severity at 6-month follow-up compared with TAU alone in patients with SUD and comorbid PTSD. We hypothesize that EMDR combined with TAU will lead to a significantly greater reduction of PTSD symptoms than TAU alone, when comparing PTSD symptoms from baseline to 6-month follow-up. As secondary outcomes, we examine the effectiveness of EMDR on substance use-related outcomes, depressive symptoms, dissociative symptoms, emotion dysregulation and quality of life. If this study shows that EMDR can be used effectively in patients with SUD and comorbid PTSD, the results of this RCT might encourage a more frequent use of evidence-based trauma-focused treatments for patients with SUD and comorbid PTSD.

## Methods

### Design and study setting

This study is a rater-blinded 2-arms RCT. Assessments are scheduled pre-treatment (T0), post-treatment (T1), at 3-month (T2) and at 6-month (T3) follow-up. Participants are randomly assigned to either the EMDR plus TAU group or the TAU group. Data is assessed at an inpatient rehabilitation center in Germany (AHG Clinic Dormagen). This center is specialized for the treatment of patients with SUD (primarily alcohol use disorders) and comorbid PTSD. The study is coordinated by a research team of the Center for Interdisciplinary Addiction Research (CIAR), University Medical Center Hamburg-Eppendorf, Germany. The coordinating site is responsible for the study design, the training of the personnel involved in the data collection, the preparation and supply of all study documents, study monitoring, study supervision, data management and analysis, and reporting of the study results.

### Participants

We plan to recruit 158 adult patients with SUD and comorbid PTSD attending inpatient rehabilitation treatment from September 2015 to December 2017. Inclusion criteria are (1) age between 18 and 65 years; (2) DSM-5 diagnosis of a substance use disorder [24]; (3) DSM-5 diagnosis of PTSD or subsyndromal PTSD (criteria A and B and at least one of the criteria C to E) [18]; (4) capable to comprehend and speak German; (5) informed consent to participate in the study. Exclusion criteria are (1) severe dissociative symptoms according to the Dissociative Experience Scale [25] (total score > 40); (2) acute suicidality; (3) acute psychotic symptoms; and (4) severe cognitive impairments.

### Interventions

All patients included in this study participate in the usual treatment (TAU) of the SUD rehabilitation center provided for patients with SUD and comorbid PTSD. TAU includes SUD rehabilitation and non-trauma-focused PTSD treatment. TAU is present-focused and includes the provision of knowledge, techniques and skills to better cope with PTSD symptoms and to prevent SUD relapse (psychoeducation about PTSD and SUD; resource activation, e.g. establishing positive activities; imaginative exercises [26]; acceptance- and mindfulness-oriented skills [27]; elements of Seeking Safety [28], non-trauma-focused CBT). A treatment protocol was created prior to the start of the study that defines the intervention elements of the non-trauma-focused treatment. No processing of traumatic memories takes place in the TAU condition.

All study participants receive two 90-min and two 60-min non-trauma-focused group therapy sessions per week. In addition to the non-trauma-focused group therapy, participants randomized to the TAU group additionally receive one 50-min individual non-trauma-focused therapy session per week; participants randomized to the EMDR group receive one 50-min individual EMDR session per week. During an EMDR session, the patient focuses on a traumatic experience while simultaneously focusing on an external bilateral stimulus [29]. EMDR treatment follows standard EMDR protocols for processing memories of traumatic or stressful life events [30].

### Measures

#### Diagnoses of SUD and PTSD

To assess DSM-5 diagnoses of SUD and PTSD, all patients receive a semi-structured face-to-face interview at baseline (T0). The diagnosis of SUD is confirmed using the SUD section of the International Diagnostic Checklists for DSM-IV (IDCL) [31], adapted according to the changed SUD criteria in DSM-V. The IDCL is an established method for the assessment of psychiatric diagnoses. Studies have indicated good clinical practicability and satisfactory to excellent inter-rater and test-retest reliability [31, 32].

The diagnosis of PTSD is confirmed using the Clinician-Administered PTSD Scale (CAPS) [33, 34] for DSM-V [33, 34], which is an updated version of the CAPS for DSM-IV. The CAPS is considered the gold standard for PTSD assessment [35, 36]. The psychometric properties of the CAPS-IV have been reviewed [35] and indicate excellent convergent and discriminant validity, diagnostic utility, and sensitivity to clinical change.

#### Primary outcome

The primary outcome, change from baseline in PTSD symptom severity, is assessed using the CAPS for DSM-V [33, 34]. The interviewer evaluates the severity of each of the 20 DSM-V PTSD symptoms, based on a combined evaluation of the frequency and intensity on Likert-type scales (0 = asymptomatic, 1 = mild/subthreshold, 2 = moderate PTSD/above threshold, 3 = severe/markedly increased, 4 = extreme). The 20 DSM-5 PTSD symptom severity scores are then summed up to derive a total severity score, ranging from 0 to 80. A change of 15 points on the CAPS is considered clinically significant [35].

#### Seconday outcomes

Secondary outcomes include change from baseline in severity of substance use, addiction-related problems, dissociative symptoms, depressive symptoms, emotion dysregulation and quality of life (Table 1).

**Table 1:** Measures and assessment points used in the study

Variable	Measure	Assessment method	T0 Baseline	T1 Post treatment	T2 3 months	T3 6 months
SUD diagnosis	IDCL [31]	interview	x	x	x	x
PTSD diagnosis	CAPS [33]	interview	x	x	x	x
PTSD symptoms	CAPS [33]	interview	x	x	x	x
Early traumatic experiences	CTQ [46]	self-report	x	x	x	x
Substance use	AUDIT [37]/DUDIT [40] TLFB [41]	interview	x	x	x	x
Addiction-related problems	ASI-Lite [42]	interview	x	x	x	x
Dissociative symptoms	DES [25]	self-report	x	x	x	x
Depressive symptoms	BDI-II [43]	self-report	x	x	x	x
Emotion dysregulation	DERS [44]	self-report	x	x	x	x
Quality of life	SF-12 [45]	self-report	x	x	x	x
Safety	SAE Report Form	self-report	----------------		x	x

*Notes. IDCL* International Diagnostic Checklists. *CAPS* Clinician-Administered PTSD Scale. *CTQ* Childhood Trauma Questionnaire. *AUDIT* Alcohol Use Disorders Identification Test. *DUDIT* Drug Use Disorders Identification Test. *TLFB* Timeline Follow Back. *ASI-Lite* Addiction Severity Index-Lite. *DES* Dissociative Experience Scale. *BDI-II* Beck Depression Inventory-II. *DERS* Difficulties in Emotion Regulation Scale. *SF-12* Short-Form 12-Item Health Survey. *SAE* Serious Adverse Event

Change from baseline in severity of substance use is measured by the Alcohol Use Disorder Identification Test (AUDIT) [37, 38] or the Drug Use Disorder Identification Test (DUDIT) [39, 40], respectively, depending on the type of the patients’ primary substance dependence. As additional substance-related outcomes, the mean amount of substance use per day and the number of non-consuming days within the last month is measured with the Timeline Follow Back (TLFB) [41]. Changes in addiction-related problems are assessed by the Addiction Severity Index-Lite (ASI-Lite) [42].

Change in dissociative symptoms (Dissociative Experiences Scale, DES) [25], depressive symptoms (Beck Depression Inventory-II, BDI-II) [43], emotion dysregulation (Difficulties in Emotion Regulation Scale, DERS) [44] and quality of life (Short-Form 12-Item Health Survey, SF-12) [45] are also assessed. The type and severity of early traumatic experiences are measured using the Childhood Trauma Questionnaire (CTQ) [46]. Sociodemographic data of the patients are measured by an interview using a sheet designed according to the documentation standards for the evaluation of addiction treatment [47].

Serious Adverse Events (SAEs; hospitalization, life-threatening event, disability or permanent damage, death) are measured by SAE Reporting Forms (date; initials of reporting person; participant study number and participant date of birth; SAE description; start date and duration of event; action required; action with regard to intervention; date of last intervention session; causality to intervention or assessment).

### Study procedure

**Fig. 1:**
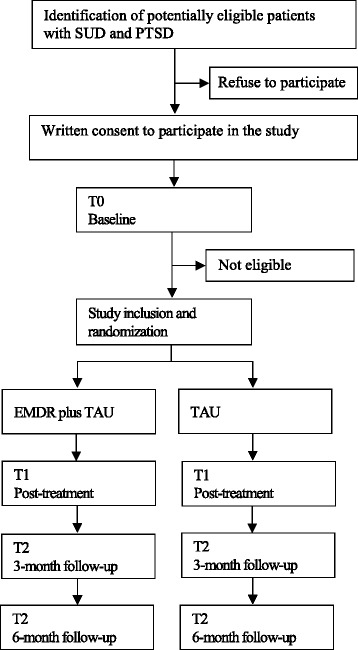
Participant flow diagram

At the patients’ admission to the rehabilitation center, a clinical psychologist informs the patient about the study and screens the patient according to the inclusion and exclusion study criteria. Patients that are potentially eligible for the study and consent to participate will receive the baseline assessment (T0) within the first days after admission (Fig. 1). If all inclusion and no exclusion criteria are fulfilled, the patient will be randomized to one of the two study arms. Additional data assessments are conducted at end of treatment (T1), as well as at 3-months and 6-months follow-up (T2 and T3, respectively). The interviews at T0 and T1 are conducted face-to-face, the interviews at T2 and T3 are conducted by telephone. To promote participant retention and complete follow-up, participants will be contacted several times between the assessments. The coordinating study team monitors the study procedure and traces back potential Serious Adverse Events during the study.

### Sample size calculation

Based on the results of the power analysis of a first RCT examining the effects of a trauma-focused PTSD treatment plus usual substance use treatment vs. usual substance use treatment ﻿alone﻿ in patients with PTSD and SUD [11], using the Clinician-Administered PTSD Scale (CAPS) as primary outcome, a sample size of 88 participants is required to achieve 80% power to detect a clinical significant difference of 15 points (SD = 25) in PTSD symptoms at 6-months follow-up, using mixed models with a .05 α-level of significance. In line with the dropout rates reported in previous intervention studies using rehabilitation inpatients [48, 49], a dropout rate of 40% is expected at 6-month follow-up. Consequently, we aim to include 158 participants in our study.

### Statistical analysis

Summary tables will be provided for all baseline, end of treatment and follow-up variables. Data will be summarized using frequency tables and descriptive statistics (mean, standard deviation, number and percentages of cases). Intention-to-treat and per-protocol analyses will be conducted for all outcomes. Missing data will be imputed using multiple imputation or a similar strategy.

To test the hypothesis that EMDR plus TAU will more greatly reduce PTSD symptoms than TAU alone, a linear mixed model will be calculated. Changes in PTSD symptom severity at 6-month follow-up as measured by the CAPS will be the primary outcome of the study and will be compared between the EMDR plus TAU and TAU group. The analysis will be adjusted for covariates that might be unbalanced between the groups (e.g., sex, type of trauma, treatment time spent in rehabilitation center), as well as for different baseline CAPS scores. The same procedure will be applied for secondary outcomes.

### Methods to protect against sources of bias

#### Randomization and allocation

All patients eligible for the study and providing written consent are randomized to an EMDR or a TAU group in a ratio of 1:1. The generation of the randomization sequence took place prior to the recruitment of participants and was conducted by a researcher of the coordinating site uninvolved in the data assessments of the study (AL). The list for the random assignment of patients to the two treatment arms was generated by the randomization software DatInf RandList Version 1.2 using permuted blocks of random sizes between 4 and 10. Each random number is separately stored in sequentially numbered, opaque and sealed envelopes. After the eligibility of a patient for the study has been ensured, the patient is included in the study and the next available randomization number is assigned to the patient in an ascending order.

#### Blinding

Study personnel involved in the data assessment of the study are blinded to the patients’ treatment assignment. The clinical psychotherapists that deliver the study treatment are not blinded and are therefore uninvolved in the patients’ data assessments related to this study. Data analysts are blinded to group allocation.

#### Treatment fidelity

All psychotherapists involved in this study regularly work as psychotherapists in the rehabilitation center at which the study is conducted. They were trained in SUD rehabilitation and non-trauma-focused PTSD treatment and completed the EMDR Basic Training program (see http://www.emdr-europe.org/info.asp?CategoryID=83). All treatments are conducted in line with the respective treatment protocols (see description of interventions). All therapists are supervised by certified EMDR consultants or trainers of EMDR Europe (LCF, PL, AH) during the treatment phase of the study.

Psychotherapists document the content of each individual treatment session in a treatment log, which is monitored by the coordinating site. Out of all individual treatment sessions, 15% randomly chosen videotaped sessions of each of the two treatment groups are rated for treatment fidelity (compliance with the treatment manual). EMDR treatment adherence is evaluated and monitored by certified EMDR consultants or trainers (LCF, PL, AH) using the EMDR Implementation Treatment Fidelity Scale [50]. Therapist adherence for individual SUD rehabilitation and non-trauma-focused PTSD treatment is evaluated and monitored over the course of study by trained raters of the coordinating site.

#### Data assessment fidelity

The personnel involved in data collection (psychology students with at least BSc level) received a 1-day training by the coordinating site prior to the start of the study. The coordinating site monitors the conduction of the data assessments and supervises (AL) the personnel involved in data collection throughout the study. 15% of the CAPS interviews are reassessed by a second interviewer to establish inter-rater reliability of the PTSD diagnosis and symptom severity. If reliability is low, the interviewers receive additional training. To improve data entry accuracy, all data sheets are entered by using a data scanner. Data entries are checked by data management personnel for correctness.

### Ethical and safety issues

The Ethics Committee of the Medical Council of Hamburg (PV4853) and the Ethics Committee of the Medical Council of Nordrhein (2015233) approved the trial prior to the start of the study. Before patients are included in the study, they are informed about the aims and the design of the study including randomization and the possibility of ending their participation at any time without disadvantages. Potential study participants must provide written informed consent before they can be included in the study. Personal information about potential and enrolled participants will be collected and stored separately from other study data and will be only accessible for the assessors that contact the participants for data assessment. The study dataset does not include personal information and will be analyzed by the coordinating study site. Throughout the study, the standard safety procedures of the inpatient rehabilitation center at which the patients of this study are treated are followed. SAEs are continuously documented throughout the study. SAEs are reported to the coordinating site within 24 h. In case of an unexpected SAE (e.g., life-threatening event, permanent damage or death) over the course of the study, the coordinating site will alert the principal investigator (IS) who will report the SAE to the local ethics committee. The ethics committee and the study team will then decide in accordance with the best interest of the patient if the study procedures are continued or terminated.

## Discussion

This is the first RCT that examines the effectiveness of EMDR combined with TAU (SUD rehabilitation and non-trauma-focused PTSD treatment), in reducing PTSD symptoms in patients with SUD and comorbid PTSD, compared with TAU alone. To test the effectiveness of EMDR within this patient group is crucial, because patients with SUD and comorbid PTSD have been excluded from most clinical trials of trauma-focused interventions for patients with PTSD so far [51]. Consequently, there is limited evidence that EDMR is effective in this patient group. As a secondary outcome, we also examine the effectiveness of EMDR on substance use. As patients may use substances to regulate PTSD-related symptoms [12, 52], it could be expected that trauma-focused treatment might also reduce SUD symptoms.

### Strengths and limitations

A strength of this study is that we will be able to include a sufficient sample size in our study to detect group differences between the EMDR plus TAU and the TAU group. However, we expect a high drop-out rate in our sample of patients with comorbid mental disorders. The expected high drop-out rate might complicate the interpretation of the study results. A strength of this study might be that the patients included in this study live in various parts of Germany, represent a great variety of their sociodemographic backgrounds and were exposed to different types of traumatic experiences. This may increase the generalizability of our results. On the other hand, we exclude patients that are younger than 18 or older than 65 years; don’t speak German; present acute suicidal, psychotic or severe dissociative symptoms; or show severely cognitive impairment. The study findings might not be generalized to these populations of patients with SUD and PTSD.

In summary, the primary aim of this study is to investigate the effectiveness of EMDR, added to usual SUD rehabilitation and non-trauma-focused PTSD treatment (TAU), in reducing PTSD symptoms, compared with TAU alone. If this RCT proves that EMDR is effective in patients with SUD and comorbid PTSD, the results of this RCT may encourage a more frequent use of evidence-based trauma-focused approaches in routine treatment of patients with SUD and comorbid PTSD.

### Trial status

We are recruiting patients and started with data collection. The first patient was enrolled in September 2015.

## Abbreviations

AE: Adverse events
ASI-Lite: Addiction Severity Index-Lite
AUDIT: Alcohol Use Disorders Identification Test
BDI-II: Beck Depression Inventory-II
CAPS: Clinician-Administered PTSD Scale
DERS: Difficulties in Emotion Regulation Scale
DES: Dissociative Experience Scale
DSM-V: Diagnostic and Statistical Manual of Mental Disorders
DUDIT: Drug Use Disorders Identification Test
EMDR: Eye Movement Desensitization and Reprocessing
IDCL: International Diagnostic Checklists
PTSD: Posttraumatic stress disorder
RCT: Randomized controlled trial
SAE: Serious Adverse Event
SD: Standard deviation
SF-12: Short-Form 12-Item Health Survey
TAU: Treatment as usual
TLFB: Timeline Follow Back
